# Bilateral Supracondylar Humerus Fracture in Pediatric after a Fall on an Outstretched Hand

**DOI:** 10.1155/2019/4893563

**Published:** 2019-06-23

**Authors:** Basam Alanazi, Jameel Fakeeha, Abdulrahman Pasha, Hussam Alqulaiti, Hani Alharbi, Jameel Mahmoud

**Affiliations:** ^1^King Saud Medical City, Saudi Arabia; ^2^Ministry of Health, Saudi Arabia

## Abstract

*Background*. Supracondylar humerus fracture (SCH) is common in the pediatric age group 5-7 years, mostly due to a fall on an outstretched hand. However, a bilateral SCH is rarely observed in this age group. Management of SCH is either surgical or conservative based on the following factors: patient age, fracture pattern and neurovascular involvement. Complications of a displaced SCH can be dramatically reduced by early surgical fixation. Acute complications include: neurovascular injury and compartment syndrome, and long term complications include: stiffness, infections and angular deformities. In this article, we present a rare case of bilateral supracondylar humerus fractures with a six-months follow-up.

## 1. Introduction

SCH is one of the most common fractures in pediatric age, representing 13.9% of all types of fractures, with a mean age 6.9 years. Male to female ratio is equal. SCH represent 60% of all pediatric elbow fractures, classically occurring as a result of fall on an outstretched hand [1].

Extension type injury is more common (95-98%) than flexion type (<5%). It frequently occurs in the non- dominant extremity [2, 3].

The frequency of neurologic deficit after a SCH in pediatric has been reported 10 to 20%; it increases with type III SCH (Gartland's classification) to 49%. Anterior interosseous nerve (AIN) most common nerve injury followed by radial nerve. Ulnar nerve injuries are commonly associated with a flexion type SCH [4].

The most common type of nerve injury is neuropraxia, which usually resolves within 8-12 weeks [4]. In addition, compartment syndrome may develop in the first 12-24 hours especially if when trauma causes vascular injury and primary swelling [5].

The distal humerus physis, in contrast to the proximal humeral physis, contributes only to 15 to 20% of the overall longitudinal growth of the humerus [6]. This is predisposed to the development of angular deformities after a SCH [6].

In most cases, accepting a fracture position in which the capitellum is posterior to the anterior humeral line on the lateral view cannot be reliably predicted to remodel, and the child is likely to permanently end up with less flexion and greater extension of the affected arm.

Modern surgical techniques (e.g., closed reduction with percutaneous pinning) have reduced this frequency of angular deformities from 58% to approximately 3% [7].

However there is no reported case of any bilateral supracondylar humerus fracture in pediatrics.

## 2. Case Presentation

A 7-year old girl presented to emergency room (ER) with bilateral elbow pain and swelling one hour after a fall from a swing on her outstretched hands. She had been previously healthy, and had no previous history of fractures. On examination, she had normal vital signs and body built. No dysmorphic features were noticed. She had bilateral elbow swelling with ecchymosis, but no wounds were noticed in her arms, and no features of compartment syndrome were observed. Distal pulses were palpable and sensory and motor examination of median, ulnar and radial nerves were normal.

**Figure 1:**
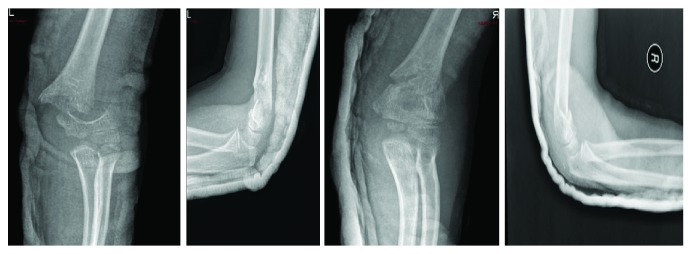
Initial X-rays. AP and lateral views of the right and left elbows.

**Figure 2:**
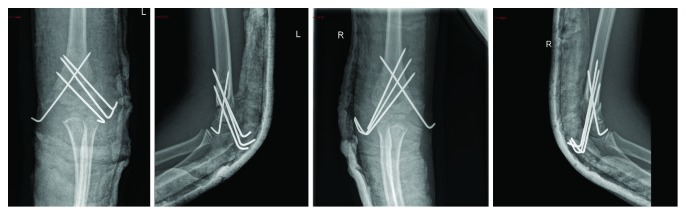
Day 1 post-op X-rays. AP and lateral views of the right and left elbows.

Both arms were splinted in ER. X-rays showed bilateral isolated Gartland IV SCH (Figure 1). She was taken to the operating room 4 hours after presentation for close reduction. The fracture was highly unstable; therefore, we decided to fix it with 4 k wires. For each side, we entered 3 wires laterally and 1 medially (Figure 2). We applied a backslap for each side. Distal pulses and neurological examination postoperatively were normal. She was maintained on a good analgesic control. She was discharged home after 2 days, during which she had underwent serial clinical examinations for compartment syndrome and X-rays to ensure correct positioning of the 4 k wires and rule out fracture displacement.

**Figure 3:**
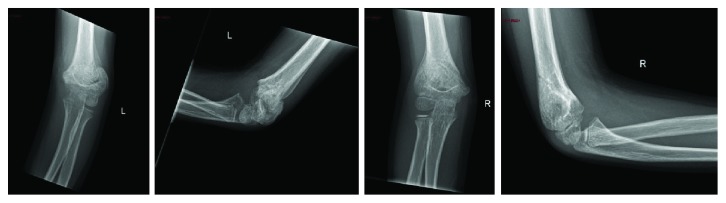
4 weeks post-op X-rays. AP and lateral views of the right and left elbows.

Patient was seen at our Orthopedic outpatient clinic 3 weeks later. She had no local infection or fracture displacement, and range of motion was decreased. Therefore, the 4 k wires were removed (Figure 3).

**Figure 4:**
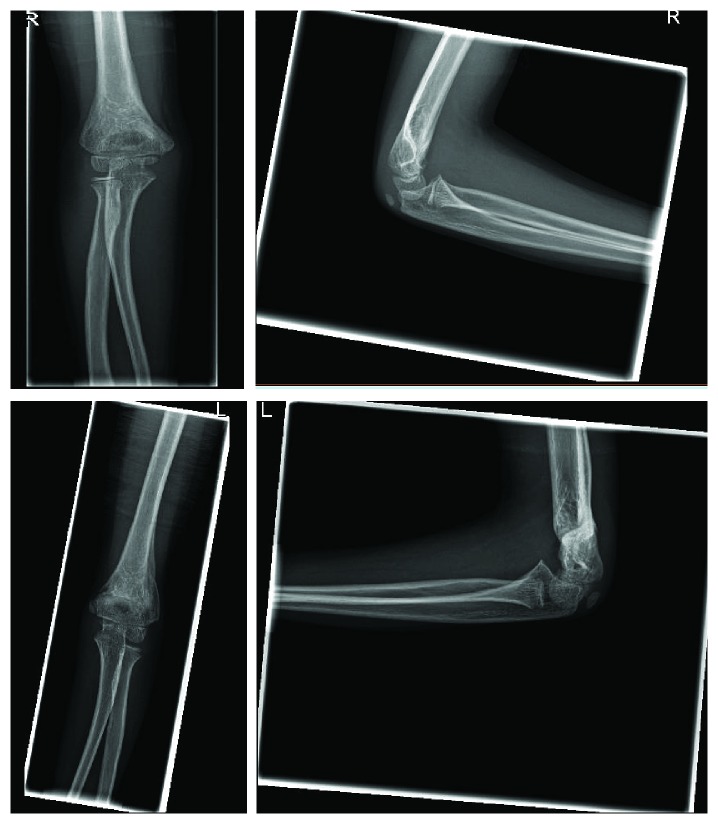
6 months post-op X-rays. AP and lateral views of the right and left elbows.

6 months follow up shows normal full range of motion of the bilateral elbow joints with completely healed fractures (Figure 4).

## 3. Discussion

Although SCH is a well-known consequence of a fall on an outstretched hand, bilateral SCH is rarely reported. A child may not provide accurate description of a fall but a high index of suspicion, a thorough clinical examination, and obtaining imaging for both elbows are essential to have accurate diagnosis.

Treatment of such cases is urgent to avoid the development of decreased range of movement at the joint when treatment is delayed. There was a few hours delay in the management of our patient; fortunately, she did not develop this complication. The risk of nerve injury during surgery is 3%, and those patients should be carefully examined pre-operatively to differentiate whether nerve injury was a result of the fall or was a complication of the surgery. High index of suspicion should be maintained for the possibility of development of compartment syndrome especially after a forearm fracture along the SCH especially with displaced fractures [8].

Operative management usually starts with a trial of close reduction. However, repetitive movement with close reduction might cause neuropraxia and joint stiffness, especially when a fracture is totally displaced. Therefore, in such cases, open reduction is recommended. In fact, open reduction technique allows obtaining an adequate anatomical reduction, which favors satisfactory functional and cosmetic outcomes, and has fewer complications than close reduction [9].

Good preoperative examination is mandatory because the risk of nerve injury of SCH fracture after operation is 3% [10].

The treatment for SCH fracture is urgent, however the delay did not cause high complications but there is a significant relationship between delay in treatment of pediatric supracondylar humeral fracture and reduction in range of movement [9, 11].

Incidence of compartment syndrome will increase if there is a fracture of the forearm along with the SCH as well as severe displaced SCH fracture so a high index of suspicion should be maintained for compartment syndrome of the arm as well as the forearm [12].

Operative management technique starting closely, however a totally displaced fracture is difficult to manage closely, if anatomic reduction can not be achieved then open reduction should be performed because repetitive manipulations could result in joint stiffness and transient neuropraxia [13].

Obtaining an adequate anatomical reduction favors excellent to good functional and cosmetic outcomes as well as fewer complications [13].

In comparing medial and lateral entry pinning technique, similar functional and radiological outcome and almost equal mechanical stability, whereas the risk of ulnar nerve injury was five times higher in medial pinning [14, 15].

The technique of fixation with K-wires is a stable and reliable methods for unstable supracondylar fracture but medial-lateral three-pin fixation is better than two pins fixation [8].

Recommended percutaneous pinning with lateral 2 divergent wires in supracondylar humerus fractures in children classified as Gartland IIB and use of crossed wires for Gartland type III or IV, using the mini-open technique for the medial wire [15].

Our case intraoperative we found it is not stable for that 4 k-wires (three lateral & one medial) inserted.

## 4. Conclusion

As we know the supracondylar humerus fracture has many complications, so if you face a case of bilateral supracondylar humerus fracture, the complications and the urgency of the management increased and good monitoring for the post op should be more closely.
